# Overexpression of CLC-3 is regulated by XRCC5 and is a poor prognostic biomarker for gastric cancer

**DOI:** 10.1186/s13045-018-0660-y

**Published:** 2018-09-14

**Authors:** Zhuoyu Gu, Yixin Li, Xiaoya Yang, Meisheng Yu, Zhanru Chen, Chan Zhao, Lixin Chen, Liwei Wang

**Affiliations:** 1 Department of Pharmacology, Medical College, Jinan University, Guangzhou, 510632 China; 2 Department of Pathophysiology, Medical College, Jinan University, Guangzhou, China; 3 Department of Clinical Oncology, The First Affiliated Hospital, Zhengzhou University, Zhengzhou, China; 4 Department of Physiology, Medical College, Jinan University, Guangzhou, 510632 China

**Keywords:** CLC-3, XRCC5, Prognosis, Biomarker, Gastric cancer

## Abstract

**Background:**

Recently, many potential prognostic biomarkers for gastric cancer (GC) have been identified, but the prognosis of advanced GC patients remains poor. Chloride channels are promising cancer biomarkers, and their family member chloride channel-3 (CLC-3) is involved in multiple biological behaviors. However, whether CLC-3 is a prognostic biomarker for GC patients is rarely reported. The molecular mechanisms by which CLC-3 is regulated in GC are unclear.

**Methods:**

The expression of CLC-3 and XRCC5 in human specimens was analyzed using immunohistochemistry. The primary biological functions and pathways related to CLC-3 were enriched by RNA sequencing. A 5′-biotin-labeled DNA probe with a promoter region between − 248 and + 226 was synthesized to pull down CLC-3 promoter-binding proteins. Functional studies were detected by MTS, clone formation, wound scratch, transwell, and xenograft mice model. Mechanistic studies were investigated by streptavidin-agarose-mediated DNA pull-down, mass spectrometry, ChIP, dual-luciferase reporter assay system, Co-IP, and immunofluorescence.

**Results:**

The results showed that CLC-3 was overexpressed in human GC tissues and that overexpression of CLC-3 was a poor prognostic biomarker for GC patients (*P* = 0.012). Furthermore, higher expression of CLC-3 was correlated with deeper tumor invasion (*P* = 0.006) and increased lymph node metastasis (*P* = 0.016), and knockdown of CLC-3 inhibited cell proliferation and migration in vitro. In addition, X-ray repair cross-complementing 5 (XRCC5) was identified as a CLC-3 promoter-binding protein, and both CLC-3 (HR 1.671; 95% CI 1.012–2.758; *P* = 0.045) and XRCC5 (HR 1.795; 95% CI 1.076–2.994; *P* = 0.025) were prognostic factors of overall survival in GC patients. The in vitro and in vivo results showed that the expression and function of CLC-3 were inhibited after XRCC5 knockdown, and the inhibition effects were rescued by CLC-3 overexpression. Meanwhile, the expression and function of CLC-3 were promoted after XRCC5 overexpression, and the promotion effects were reversed by the CLC-3 knockdown. The mechanistic study revealed that knockdown of XRCC5 suppressed the binding of XRCC5 to the CLC-3 promoter and subsequent promoter activity, thus regulating CLC-3 expression at the transcriptional level by interacting with PARP1.

**Conclusions:**

Our findings indicate that overexpression of CLC-3 is regulated by XRCC5 and is a poor prognostic biomarker for gastric cancer. Double targeting CLC-3 and XRCC5 may provide the promising therapeutic potential for GC treatment.

**Electronic supplementary material:**

The online version of this article (10.1186/s13045-018-0660-y) contains supplementary material, which is available to authorized users.

## Background

Gastric cancer (GC), the second leading cause of cancer-related death worldwide, is characterized by advanced clinical stages at diagnosis and poor survival rates [1, 2]. In 2012, GC accounted for 6.8% of global cancer incidence and 8.8% of cancer mortality worldwide [3]. GC development is a complicated multistep process, influenced by a *Helicobacter pylori* infection, host genetic susceptibility, and other environmental factors [4]. Achieving a detailed understanding of the molecular pathogenesis associated with GC will be critical to improving patient outcomes. Recently, many potential prognosis biomarkers for GC have been identified, including BMI1, Ezh2, and LINC00261 [5–8]. However, the prognosis of advanced GC remains poor. Therefore, the identification of further biomarkers for therapeutic purposes in GC is imperative.

Chloride channels are promising cancer biomarkers by mediating a multitude of biological functions [9]. Chloride channel-3 (CLC-3), a member of the voltage-gated chloride channel family, mainly mediates the extra- and intracellular ion homeostasis and acidification of intracellular compartments. Recent studies have revealed that CLC-3 participates in the processes of cell volume regulation, proliferation, and migration, particularly in glioma and prostate cancer cells [10–12]. Our previous studies have indicated that CLC-3 is overexpressed in nasopharyngeal carcinoma cells and plays roles in controlling cell proliferation [13]. Moreover, suppression of CLC-3 expression reduces the migration of nasopharyngeal carcinoma, hepatocellular carcinoma, and cervical carcinoma cells [14–16]. Therefore, CLC-3 may play key roles in tumor development. However, whether CLC-3 is a prognostic biomarker for GC patients is rarely reported. The molecular mechanisms by which CLC-3 is regulated in GC are unclear.

Our present study indicated that overexpression of CLC-3 was a poor prognostic marker for GC patients and that cell proliferation and migration were the primary biological functions of CLC-3 in GC cells. Moreover, XRCC5, a subunit of the Ku heterodimer protein [17, 18], was identified to be a promoter-binding protein of CLC-3. As a key mediator of DNA recombination, chemotherapy resistance, and chromosome stability maintenance [19–21], XRCC5 has elevated expression in a variety of tumors [22–24]. However, little is known about the expression of XRCC5 in GC. In this study, we showed that XRCC5 was highly expressed in GC. Importantly, the expression and function of CLC-3 were regulated by XRCC5 in vivo and in vitro, and both CLC-3 and XRCC5 were prognostic factors of overall survival in GC patients. The relative expressions of CLC-3 and XRCC5 could determine the further prognosis of GC patients.

## Methods

### Patient samples

Paraffin-embedded tumor tissues and adjacent normal tissues were obtained from 90 patients diagnosed with gastric adenocarcinoma between May 2007 and February 2008 at the First Affiliated Hospital of Zhengzhou University. Medical records of all patients provided information about age, gender, pathological grade, and TNM stage. The patients were followed up for 8 years. Written informed consent was obtained from each patient involved in the study, and the study was approved by the Ethics Committee of Zhengzhou University.

### Cell culture and stable cell line construction

Human GC cell lines (SGC-7901, BGC-823, and AGS) and human normal gastric epithelial (GES-1) cells were obtained and authenticated from the Cell Bank of the Chinese Academy of Sciences (Shanghai, China). All cells were cultured in RPMI 1640 medium supplemented with 10% fetal bovine serum. Lentiviruses for XRCC5 knockdown (shXR-1 and shXR-2), XRCC5 overexpression (XRCC5), CLC-3 knockdown (shCLC-3), and CLC-3 overexpression (CLC-3) were purchased from GenePharma (Shanghai, China). SGC-7901 and BGC-823 cells were used to establish stable cell lines via selection with 1 μg/ml puromycin for 4 weeks. Negative control shRNA cells (sh-NC) and empty vector-transfected cells (vector) were established as controls.

### Streptavidin-agarose-mediated DNA pull-down assay

A biotin-labeled double-stranded oligonucleotide probe for the − 248 to + 226 fragment of the CLC-3 promoter sequence was synthesized by Ruibiotech Co. (Beijing, China). Briefly, 1 mg of nuclear protein extract was mixed and incubated with 10 μg of probe and 100 μl of streptavidin-agarose beads (Sigma, St Louis, MO). The mixtures were then centrifuged at 800×*g*, resuspended in 30 μl of loading buffer, and boiled at 100 °C for 5 min. The collected samples containing the bound proteins were separated by SDS-PAGE for further silver staining or Western blot analysis.

### Silver staining and mass spectrometry

After electrophoresis of the samples containing the bound proteins, the protein gel was immersed in a stationary liquid with 10% acetic acid, 50% ethanol, and 40% water at room temperature on a shaker overnight. The protein bands were visualized with a fast silver staining kit (Beyotime, Shanghai, China) and analyzed using MS by Honortech (Beijing, China).

### Chromatin immunoprecipitation assay

The chromatin immunoprecipitation (ChIP) procedure was performed as illustrated in the ChIP kit (cat# 9002S, Cell Signaling Technology, Danvers, MA). Briefly, the tested cell lines were fixed with formaldehyde, and cross-linking was performed by adding glycine. The samples were placed on ice and sonicated to separate the DNA into 100 to 1000-bp fragments. Then, they were incubated with antibodies at a dilution of 1:50 overnight, followed by incubation with protein G agarose beads at 4 °C overnight. Next, the bound DNA-protein mixtures were eluted, and cross-linking was reversed after several washes. The DNA fragments were then purified, and PCR was performed with CLC-3 primers purchased from GeneCopoeia (cat# HQP001983, Rockville, MD, USA) to amplify a 102-bp segment. The PCR products were separated on 2% agarose gels and visualized on a UV transilluminator.

### Dual-luciferase reporter assay

The pGL4.13 vector was used as a positive control for the luciferase reporter system. Fragments including the CLC-3 promoter region were inserted between the HindІІІ and KpnI sites of the pGL4.10 luciferase vector (Promega, Madison, WI). Primer pairs were designed for the truncated promoter regions, as shown in Additional file 1: Table S1. Briefly, stable cell lines were plated in 96-well plates and transfected with luciferase plasmid. To normalize the transfection efficiency, the cells were co-transfected with the Renilla luciferase control reporter pRL-TK vector by using EndoFectin™ Max (GeneCopoeia, Inc.). Luciferase activity was detected using the Dual-Luciferase® Reporter Assay System (Promega) after 48 h.

### RNA extraction and quantitative RT-PCR

Total RNA was extracted from cells using a RaPure Total RNA Micro Kit (Magen, Guangzhou, China). Endogenous cDNA was obtained by the ReverTra Ace qPCR RT Master Mix kit (Toyobo, Shanghai, China). Primers for CLC-3 (cat# HQP001983), XRCC5 (cat# HQP018568), and GAPDH (cat# HQP006940) were obtained from GeneCopoeia Inc. Finally, qRT-PCR was performed with SYBR® Green Real-time PCR Master Mix (Toyobo) in a Bio-Rad CFX96 PCR system. Relative RNA levels were calculated as the fold changes with the 2^−ΔΔCT^ formula.

### Co-immunoprecipitation (Co-IP) assay and Western blot analysis

Protein extracts were prepared and incubated with antibodies against XRCC5 or IgG for 24 h on a rotating wheel. Then, Protein A/G plus-Agarose beads (Santa Cruz, Dallas TX, USA) were added and incubated for another 24 h. After the beads were boiled, the precipitated proteins were separated by SDS-PAGE and transferred to PVDF membranes for further analysis. For Western blot (WB) analysis, equal amounts of proteins from the lysates were separated and transferred. The membranes were blocked with 5% nonfat milk for 2 h and then incubated with antibodies. The protein bands were finally detected by enhanced chemiluminescence. The density of the protein bands was quantified by ImageJ software (National Institutes of Health, Bethesda, MD) and normalized to GAPDH. Relative protein levels were calculated as the density ratios of interest protein to GAPDH. All antibodies used for WB were purchased from Cell Signaling Technology (Danvers, MA, USA).

### MTS assay and clone formation assay

Cell proliferation was determined by MTS assays (Promega, Madison, WI). Different stable cell lines and stable cell lines transfected with PARP1 siRNA (GenePharma, Shanghai, China) were seeded at a density of 5000 cells per well in 96-well plates. At the time points of 24 h, 48 h, and 72 h after seeding, the cells were respectively incubated with MTS for 40 min, and the optical density (OD) was then detected with a microplate reader. For the clone formation assay, cells were seeded at a density of 500 cells per well in 6-well plates and cultured for 2 weeks. The formative colonies were then fixed with formalin and stained by crystal violet. The number of clones was counted by Image-Pro Plus 6.0 software.

### Wound scratch assay and transwell assay

Cell migration ability was examined by the wound scratch assay. Briefly, cells were cultured in 6-well plates until reaching confluence and then were scratched with a 10-μl pipette tip. The gap widths at 0 h (w1) and at 36 or 48 h (w2) were measured, and the relative migration rate was calculated as (w1 − w2)/w1 × 100%. Transwell assay was performed with Boyden chambers containing 24-well transwell plates (BD, Franklin Lakes, USA). Homogeneous single-cell suspensions were added to the upper chambers coated with Matrigel. After 24 h, invaded cells on the bottom of the chambers were stained with crystal violet and counted in five random fields.

### RNA sequencing

Briefly, samples (SGC-7901 cells transfected with control or CLC-3 shRNA) were used to extract total RNA for RNA-seq loading and quality control. Differential gene expression (DGE) RNA-seq was then performed, and 50-bp paired-end reads were finally produced (RiboBio, Guangzhou, China). NCBI Sequence Read Archive (SRA) sequencing data were submitted under accession number SRP135951.

### In vivo tumor model

All animal experimental procedures were approved by the Animal Care and Use Committee of Jinan University. Approximately 2 × 10^6^ cells in 100 μl of PBS were subcutaneously injected. Tumor volumes were recorded every 4 days and calculated according to the equation of volume = (width^2^ × length)/2. After 4 weeks, the tumor xenografts were harvested, weighed, and processed for immunohistochemistry (IHC) staining.

### Immunofluorescence and immunohistochemistry

Cells were first seeded onto coverslips in a 6-well plate. Subsequently, the cells were fixed with 4% paraformaldehyde, permeabilized with 0.5% Triton-X, and blocked with bovine serum albumin (BSA). The coverslips were then incubated with primary antibodies at a dilution of 1:200 overnight. After washing, the coverslips were incubated with secondary antibodies and stained with 4,6-diamidino-2-phenylindole (DAPI). The immunofluorescence images were captured by a confocal microscope (Olympus, Japan). For IHC staining, the paraffin-embedded sections were incubated with anti-XRCC5 and anti-CLC-3 primary antibodies at a dilution of 1:100 overnight. After washing, the sections were incubated with horseradish peroxidase-conjugated anti-goat antibodies and stained with 3,5-diaminobenzidine (DAB). The percentage of stained cells (0–100%) was multiplied by the staining intensity (0, 1, 2, or 3) to produce the final IHC scores (0–300), of which 100 or higher was considered to indicate high XRCC5 expression, and 60 or higher was considered to indicate high CLC-3 expression.

### Statistical analysis

Statistical analyses were performed using SPSS statistical software. All data were presented as the mean ± SD. The significance of difference was assessed by *t* tests or variance analysis. Correlations between XRCC5 and CLC-3 expression were assessed using Spearman rank correlation analysis, and overall survival curves were assessed using Kaplan-Meier analysis. Multivariate cumulative survival analysis was conducted with the Cox regression model. The *P* values less than 0.05 were considered statistically significant.

## Results

### Overexpression of CLC-3 was a poor prognostic biomarker for GC patients, and CLC-3 knockdown inhibited cell proliferation and migration in vitro

**Fig. 1:**
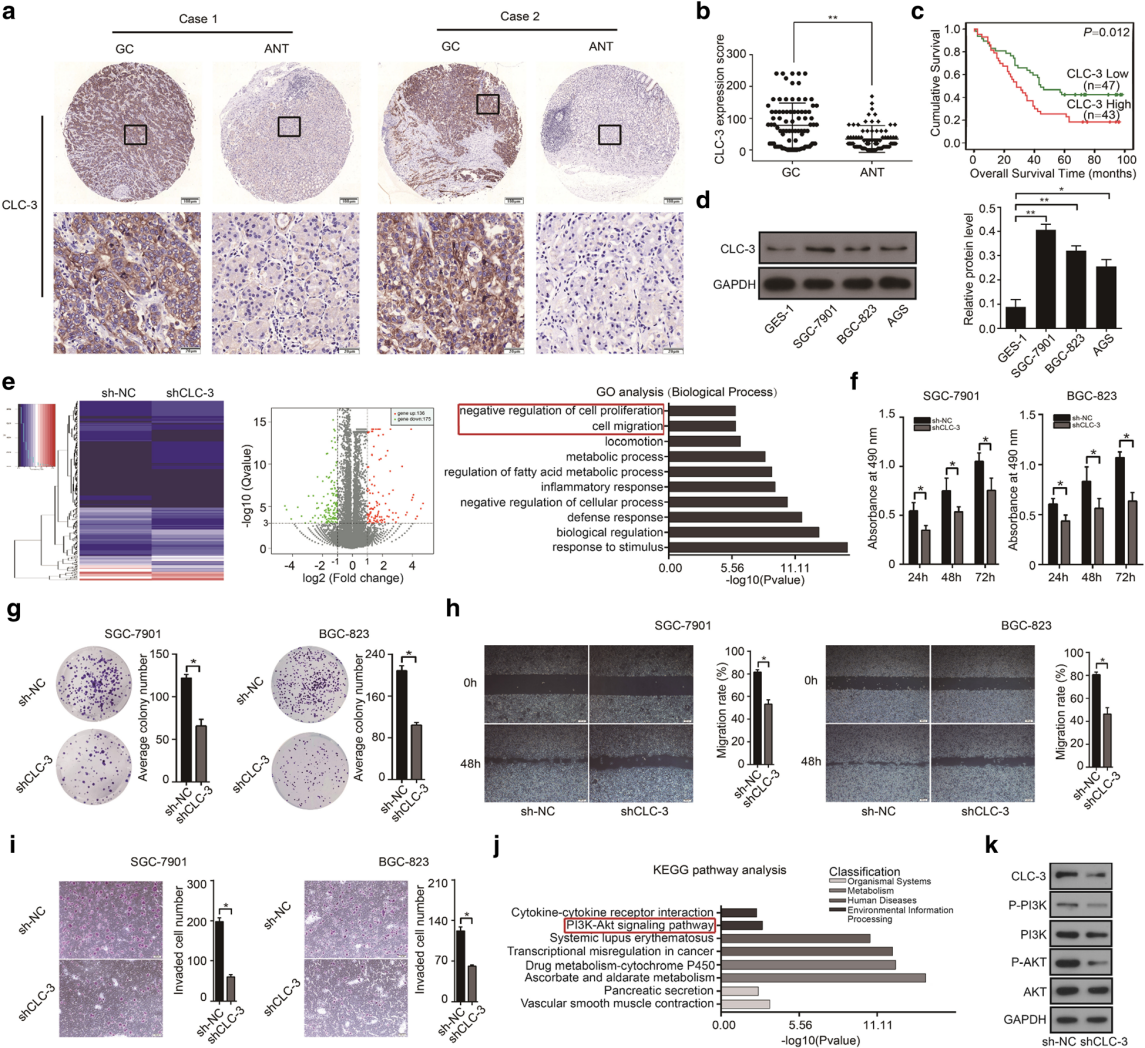
Overexpression of CLC-3 was a poor prognostic biomarker for GC patients, and CLC-3 knockdown inhibited cell proliferation and migration in vitro. **a** Representative images of CLC-3 expression in GC tissues and adjacent normal tissues (ANT). **b** The expression of CLC-3 in 90 GC tissues was higher than that in ANT. **c** High expression of CLC-3 was associated with poor prognosis in GC patients. **d** The basic protein expression of CLC-3 in human normal gastric epithelial cells (GES-1) and human GC cell lines (SGC-7901, BGC-823, and AGS). **e** The heatmap and volcano plot of RNA sequencing were constructed after the CLC-3 knockdown. Cell proliferation and migration were identified as the primary biological functions of CLC-3 according to the Gene Ontology (GO) analysis. **f**, **g** Knockdown of CLC-3 inhibited the proliferation and clonogenicity of SGC-7901 and BGC-823 cells (*n* = 3). **h**, **i** Knockdown of CLC-3 inhibited the migration and invasion of SGC-7901 and BGC-823 cells (*n* = 3). **j** The PI3K/Akt signaling pathway was enriched according to the Kyoto Encyclopedia of Genes and Genomes (KEGG) pathway analysis of CLC-3 knockdown. **k** Knockdown of CLC-3 reduced the levels of key targets in the PI3K/Akt signaling pathway. *P < 0.05, **P < 0.01

To confirm whether CLC-3 was a potential cancer biomarker for GC, in this study, we first examined the expression of CLC-3 in 90 paraffin-embedded GC tumor tissues and adjacent normal tissues (ANT) by IHC analysis. Significantly, the expression of CLC-3 was higher in GC tissues than that in ANT. Moreover, CLC-3 was mainly localized to the cell membrane and cytoplasm (Fig. 1a, b). Next, we analyzed the effect of CLC-3 expression on the cumulative survival rate of these 90 GC patients. Kaplan-Meier analysis showed that high expression (IHC score ≥ 60) of CLC-3 predicted poor survival outcome, indicating that CLC-3 overexpression was a poor prognostic biomarker for GC patients (*P* = 0.012, Fig. 1c). Furthermore, we examined the relationship between the CLC-3 expression and the clinicopathological characteristics in GC patients. As shown in Table 1, higher CLC-3 expression was correlated with deeper tumor invasion (*P* = 0.006), increased lymph node metastasis (*P* = 0.016), and later clinical staging (*P* = 0.015).

**Table 1:** Correlations between CLC-3 expression and clinicopathological characteristics of GC patients

Variables	*N*	CLC-3		*χ* ^2^	*P* value
		Low expression	High expression		
Age					
≤ 70	55	29	26	0.014	0.904
> 70	35	18	17		
Gender					
Male	70	35	35	0.623	0.430
Female	20	12	8		
Pathological grade					
I/II	24	15	9	1.386	0.239
III/IV	66	32	34		
Depth of invasion					
T1/T2	11	10	1	7.517	0.006
T3/T4	79	37	42		
Lymph node metastases					
N0	23	17	6	5.826	0.016
N1/N2/N3	67	30	37		
Distant metastasis					
M0	86	45	41	0.009	0.927
M1	4	2	2		
TNM stage					
I/II	37	25	12	5.930	0.015
III/IV	53	22	31		

*CLC-3* chloride channel-3, *GC* gastric cancer

We further investigated the role of CLC-3 in vitro. Basic protein expression of CLC-3 in human normal gastric epithelial cells (GES-1) and human GC cell lines (SGC-7901, BGC-823, and AGS) was detected by WB. The expression of CLC-3 was also increased in GC cell lines compared to that in normal cells (Fig. 1d). To identify the biological functions of CLC-3 in GC cells, the primary biological processes participated by CLC-3 were analyzed by RNA sequencing in SGC-7901 cells. The heatmap and volcano plot revealed that 136 and 175 genes (upregulated and downregulated, respectively) were differentially expressed after the CLC-3 knockdown. By Gene Ontology (GO) analysis of these differential genes, cell proliferation and migration were identified as the primary biological functions of CLC-3 (Fig. 1e). We then validated the effect of CLC-3 knockdown on cell proliferation and migration. Our results showed that knockdown of CLC-3 inhibited the proliferation of GC cells at different time points (Fig. 1f). Next, clone formation assay showed that knockdown of CLC-3 attenuated the clonogenicity of GC cells (Fig. 1g). Furthermore, the scratch assay and transwell assay indicated that knockdown of CLC-3 impaired the migration rate and number of invaded GC cells (Fig. 1h, i). To study the pathways related to CLC-3 in GC cells, Kyoto Encyclopedia of Genes and Genomes (KEGG) pathway enrichment was performed by RNA sequencing in SGC-7901 cells, and the PI3K/Akt signaling pathway was enriched as the primary pathway after CLC-3 knockdown (Fig. 1j). We then verified that knockdown of CLC-3 reduced the levels of key targets in the PI3K/Akt signaling pathway, which revealed that CLC-3 knockdown inhibited this pathway (Fig. 1k). Altogether, our results proved that as a prognostic biomarker for GC, CLC-3 also had important functions in vitro.

### XRCC5 was identified as a CLC-3 promoter-binding protein, and both CLC-3 and XRCC5 were prognostic factors of overall survival in GC patients

**Fig. 2:**
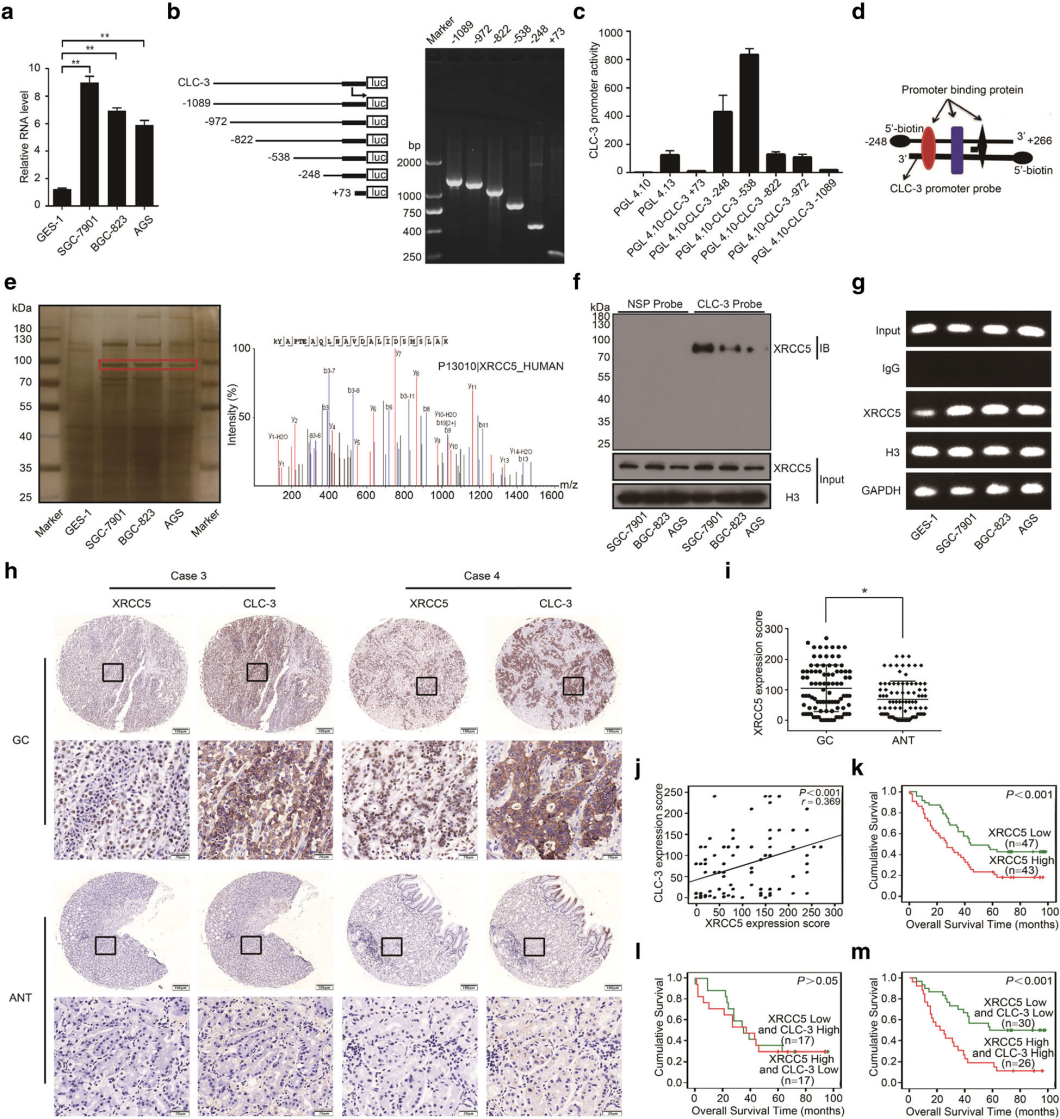
XRCC5 was identified as a CLC-3 promoter-binding protein, and both CLC-3 and XRCC5 were prognostic factors of the overall survival in GC patients. **a** The basic RNA expression of CLC-3 in cell lines (*n* = 3). **b** Truncated fragments of the CLC-3 gene promoter were designed and amplified by PCR to construct dual-fluorescence reporter plasmids. **c** The promoter activity of various reporter plasmids was measured by dual-luciferase reporter assay (*n* = 3). **d** A 5′-biotin-labeled DNA probe with a promoter region between − 248 and + 226 was synthesized. **e** Potential CLC-3 promoter-binding proteins in nuclear protein extracts were pulled down using the synthesized probe. After SDS-PAGE and silver staining, the target protein band was observed (indicated with the red rectangle), which was significantly enriched in GC cells and finally analyzed by mass spectrometry. **f** Binding between XRCC5 and the CLC-3 promoter was detected by WB in the nuclear protein/DNA complex using a synthesized probe or nonspecific probe (NSP). **g** Binding of XRCC5 to the CLC-3 DNA was confirmed by ChIP assay. The PCR products were separated on 2% agarose gels. **h** Representative images of XRCC5 and CLC-3 expression in GC tissues and ANT. **i** The expression of XRCC5 in 90 GC tissues was higher than that in ANT. **j** The expression of XRCC5 and CLC-3 was positively correlated in GC tissues. **k** High expression of XRCC5 predicted a poor prognosis for GC patients. **l** There was no difference in overall survival between GC patients with high CLC-3 and low XRCC5 levels and GC patients with high XRCC5 and low CLC-3 levels. **m** GC patients with high expression of XRCC5 and CLC-3 had the worst prognosis. *P < 0.05, **P < 0.01

To explore the molecular mechanism of CLC-3 overexpression in GC cells, we further detected the basic RNA expression of CLC-3 in the above cell lines. The RNA level of CLC-3 was also elevated in GC cells compared to that in normal cells (Fig. 2a), suggesting that the CLC-3 overexpression might be regulated by some tumor-specific factors at the transcriptional level. To study the transcriptional regulatory mechanism of CLC-3 overexpression, a series of truncated CLC-3 gene promoter fragments were amplified by PCR and cloned into the pGL4.10-basic vector to construct dual-fluorescence reporter plasmids. The results of agarose gel electrophoresis indicated that each truncated fragment had the correct size in accordance with our designed sequence (Fig. 2b). Dual-luciferase reporter assays revealed that all reporter plasmids exhibited promoter activity in SGC-7901 cells, while the pGL4.10-CLC-3 − 248 plasmid showed higher promoter activity combined with shorter sequence length, indicating that a 5′-biotin-labeled DNA probe with a promoter region between − 248 and + 226 was optimal to pull down CLC-3 promoter-binding proteins (Fig. 2c, d).

We synthesized and incubated this probe with nuclear protein extracts to pull down potential CLC-3 promoter-binding proteins. After SDS-PAGE and silver staining, the target protein band (at almost 86 kDa) was observed, and the amount of this protein band was significantly enriched in GC cells compared with normal cells (Fig. 2e, left; red rectangle). This protein band was then excised and analyzed by MS. With the best peptide-spectrum sequence of KYAPTEAQLNAVDALIDSMSLAK, XRCC5 was identified as a candidate binding to the CLC-3 promoter (Fig. 2e, right). To ascertain the binding between XRCC5 and the CLC-3 promoter, we pulled down the nuclear protein/DNA complex in GC cells using synthesized DNA probe or nonspecific probe (NSP) and validated their binding by WB (Fig. 2f). Furthermore, ChIP assays were performed to further confirm the binding of XRCC5 protein to CLC-3 DNA. We found that XRCC5 bound the CLC-3 DNA in all cells, while the smallest amount of binding was observed in normal cells (Fig. 2g).

We then explored the clinicopathologic significance of CLC-3 and XRCC5 in GC patients. IHC analysis revealed that XRCC5 was localized to the nucleus, and the expression of both CLC-3 and XRCC5 was higher in GC tissues than that in ANT. Moreover, by observing IHC images of the same site, we found that patients with strong XRCC5 staining tended to have strong CLC-3 staining (Fig. 2h, i). The Spearman rank correlation analysis showed that the expression of CLC-3 and XRCC5 was positively correlated (*r* = 0.369, *P* < 0.001) (Fig. 2j). Next, the Kaplan-Meier survival analysis revealed that high expression (IHC score ≥ 100) of XRCC5 predicted a poor prognosis for GC patients (*P* < 0.001, Fig. 2k). In addition, higher XRCC5 expression was correlated with deeper tumor invasion (*P* = 0.036) and later clinical staging (*P* = 0.045) (Table 2). Importantly, compared to those with low CLC-3 and high XRCC5 levels, the GC patients with high CLC-3 and low XRCC5 levels did not present a difference in overall survival (*P* > 0.05, Fig. 2l). However, patients with high expression of XRCC5 and CLC-3 had the worst prognosis (*P* < 0.001, Fig. 2m). To further check the prognostic value of CLC-3 and XRCC5, multivariate analysis was used to investigate the correlation between cumulative overall survival rates and clinicopathological characteristics. As shown in Table 3, four factors, including depth of invasion (hazard rate (HR) = 1.883; 95% CI 1.135–3.122; *P* = 0.014), TNM stage (HR = 2.349; 95% CI 1.342–4.114; *P* = 0.003), XRCC5 expression (HR = 1.795; 95% CI 1.076–2.994; *P* = 0.025), and CLC-3 expression (HR = 1.671; 95% CI 1.012–2.758; *P* = 0.045), were associated with the clinical outcomes of GC patients. Our results suggested that both CLC-3 and XRCC5 were prognostic factors of overall survival in GC patients.

**Table 2:** Correlations between XRCC5 expression and clinicopathological characteristics of GC patients

Variables	*N*	XRCC5		*χ* ^2^	*P* value
		Low expression	High expression		
Age					
≤ 70	55	33	22	3.429	0.064
> 70	35	14	21		
Gender					
Male	70	34	36	1.683	0.195
Female	20	13	7		
Pathological grade					
I/II	24	9	15	2.843	0.092
III/IV	66	38	28		
Depth of invasion					
T1/T2	11	9	2	4.399	0.036
T3/T4	79	38	41		
Lymph node metastases					
N0	23	14	9	0.926	0.336
N1/N2/N3	67	33	34		
Distant metastasis					
M0	86	46	40	1.243	0.265
M1	4	1	3		
TNM stage					
I/II	37	24	13	4.025	0.045
III/IV	53	23	30		

*XRCC5* X-ray repair cross-complementing 5, *GC* gastric cancer

**Table 3:** Multivariate analysis between cumulative overall survival rates and clinicopathological characteristics of GC patients

Variables	HR (95% CI)	*P* value
Age, > 70 (vs. ≤ 70)	1.734 (0.650–4.628)	0.271
Gender, female (vs. male)	1.069 (0.589–1.940)	0.827
Pathological grade, III/IV (vs. I/II)	1.260 (0.661–2.401)	0.483
Depth of invasion, T3/T4 (vs. T1/T2)	1.883 (1.135–3.122)	0.014
Lymph node metastases, N1/N2/N3 (vs. N0)	2.251 (0.809–6.261)	0.120
Distant metastasis, M1 (vs. M0)	1.480 (0.815–2.687)	0.198
TNM stage, III/IV (vs. I/II)	2.349 (1.342–4.114)	0.003
XRCC5 expression, high (vs. low)	1.795 (1.076–2.994)	0.025
CLC-3 expression, high (vs. low)	1.671 (1.012–2.758)	0.045

*HR* hazard rate, *CI* confidence interval, *XRCC5* X-ray repair cross complementing 5, *CLC-3* chloride channel-3, *GC* gastric cancer

### The expression and function of CLC-3 were regulated by XRCC5 in vitro

**Fig. 3:**
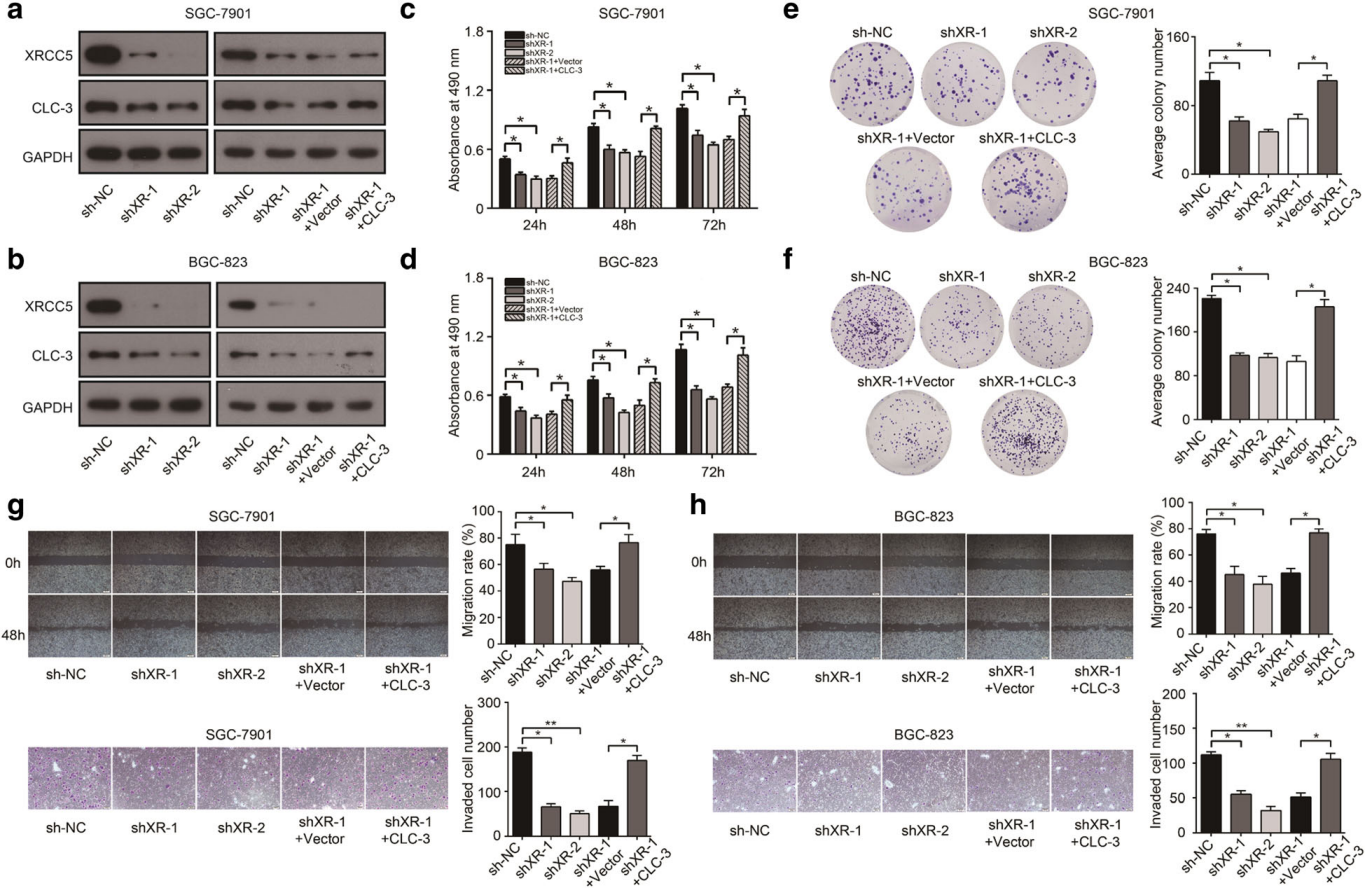
The expression and function of CLC-3 were inhibited after XRCC5 knockdown. **a**, **b** The expression of CLC-3 was inhibited after XRCC5 knockdown in SGC-7901 and BGC-823 cells, and the inhibition effect was rescued by CLC-3 overexpression. **c**–**f** The proliferation and clonogenicity of SGC-7901 and BGC-823 cells were attenuated after XRCC5 knockdown, and the attenuation effects were rescued by CLC-3 overexpression (*n* = 3). **g**, **h** The migration and invasion of SGC-7901 and BGC-823 cells were inhibited after XRCC5 knockdown, and the inhibition effects were similarly rescued by CLC-3 overexpression (*n* = 3). *P < 0.05, **P < 0.01

To study the relationship between XRCC5 and CLC-3 in vitro, we established stable XRCC5-knockdown cell lines (SGC-7901 and BGC-823) and corresponding rescue models by transfecting lentivirus of CLC-3 overexpression. WB revealed that the expression of CLC-3 was inhibited after XRCC5 knockdown in SGC-7901 and BGC-823 cells, and the inhibition effect was rescued by CLC-3 overexpression (Fig. 3a, b). According to the primary biological functions of CLC-3, we investigated the effects of XRCC5 knockdown on cell proliferation and migration. The proliferation of GC cells was attenuated after XRCC5 knockdown, and the attenuation effect was rescued by CLC-3 overexpression (Fig. 3c, d). Furthermore, clone formation assay showed that knockdown of XRCC5 attenuated the cell clonogenicity. Similarly, the attenuation effect was rescued by CLC-3 overexpression (Fig. 3e, f). Next, scratch assay and transwell assays revealed that the migration and invasion of SGC-7901 and BGC-823 cells were also inhibited after XRCC5 knockdown, and the inhibition effects were rescued by CLC-3 overexpression (Fig. 3g, h).

**Fig. 4:**
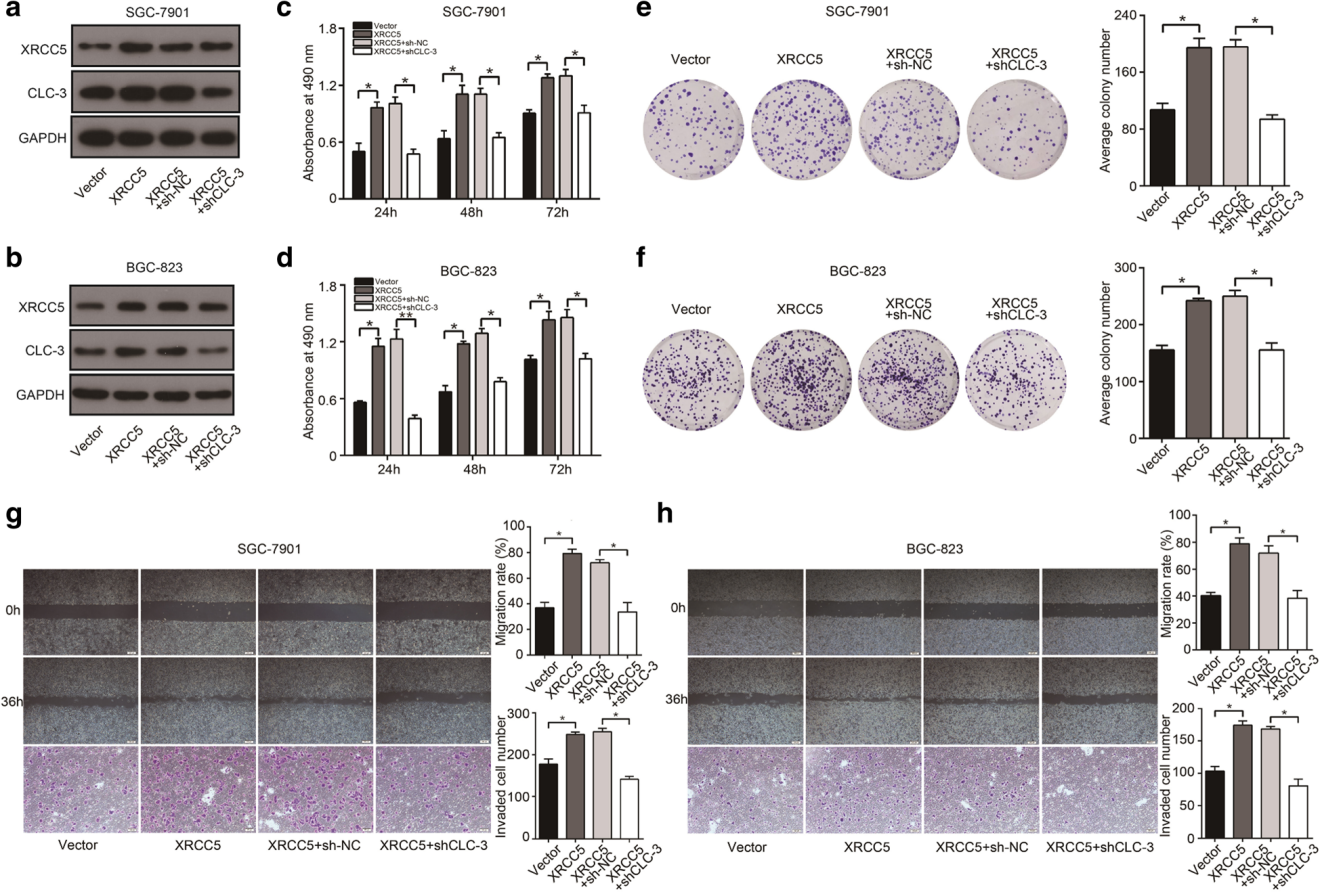
The expression and function of CLC-3 were promoted after XRCC5 overexpression. **a**, **b** The expression of CLC-3 in SGC-7901 and BGC-823 cells was increased after XRCC5 overexpression, and the increase effects could be reversed by the CLC-3 knockdown. **c**–**f** The proliferation and clonogenicity of SGC-7901 and BGC-823 cells were promoted after XRCC5 overexpression, and these promotion effects were reversed by CLC-3 knockdown (*n* = 3). **g**, **h** The migration and invasion of SGC-7901 and BGC-823 cells were enhanced by XRCC5 overexpression, and the enhancement effects were similarly reversed by CLC-3 knockdown (*n* = 3). *P < 0.05, **P < 0.01

To further evaluate whether CLC-3 was the target of XRCC5, we established stable cell lines with XRCC5 overexpression and corresponding reverse models by transfecting lentivirus of CLC-3 knockdown. The protein expression of CLC-3 in SGC-7901 and BGC-823 cells was increased after XRCC5 overexpression, and the increase effects could be reversed by CLC-3 knockdown (Fig. 4a, b). We then found that XRCC5 overexpression promoted the cell proliferation and clonogenicity, and these promotion effects could be reversed by CLC-3 knockdown (Fig. 4c–f). Scratch and transwell assays indicated that the migration and invasion of GC cells were enhanced by XRCC5 overexpression. Similarly, these enhancement effects were reversed by CLC-3 knockdown (Fig. 4g, h). These findings confirmed that the expression and function of CLC-3 were regulated by XRCC5 in vitro.

### The expression of CLC-3 was regulated at the transcriptional level by XRCC5 interacting with PARP1

**Fig. 5:**
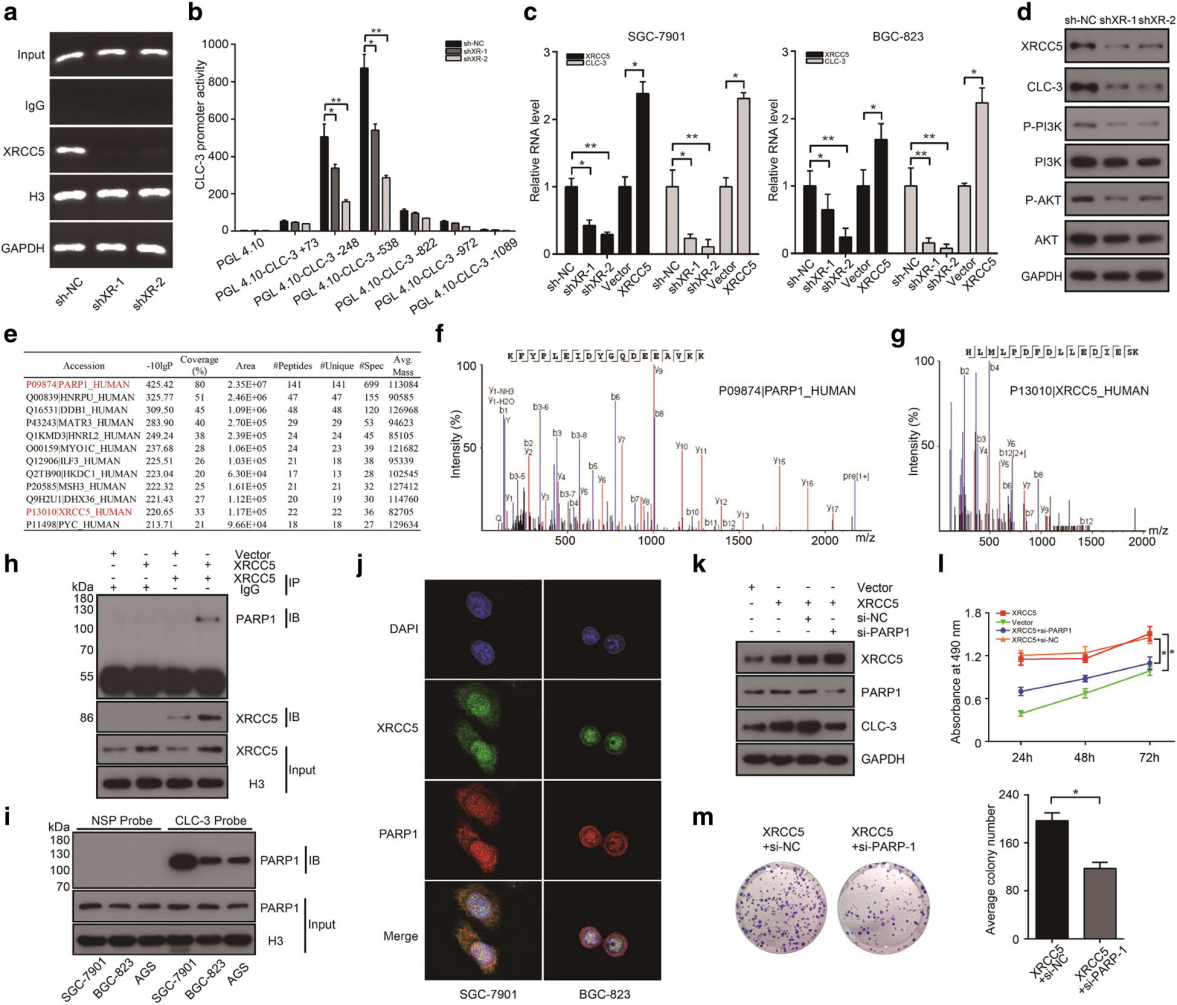
The expression of CLC-3 was regulated at the transcriptional level by XRCC5 interacting with PARP1. **a** The binding of XRCC5 to the CLC-3 DNA in SGC-7901 cells was suppressed after XRCC5 knockdown. **b** Knockdown of XRCC5 impaired the promoter activities of the pGL4.10-CLC-3 − 248 and pGL4.10-CLC-3 − 538 reporter plasmids in SGC-7901 cells (*n* = 3). **c** The RNA level of CLC-3 was reduced after XRCC5 knockdown and increased after XRCC5 overexpression (*n* = 3). **d** Knockdown of XRCC5 decreased the levels of key targets in the PI3K/Akt signaling pathway by downregulating CLC-3 in SGC-7901 cells. **e**–**g** PARP1 was identified as an interaction partner of XRCC5 by IP and MS in nuclear protein extracts of SGC-7901 cells. **h** The interaction between XRCC5 and PARP1 was confirmed by Co-IP in stable SGC-7901 cells with XRCC5 overexpression. **i** Binding between PARP1 and the CLC-3 promoter was detected by WB in the nuclear protein/DNA complex using synthesized probe or NSP. **j** The co-localization of XRCC5 and PARP1 was observed in the nucleus by confocal microscopy analysis. **k** Overexpression of XRCC5 increased the expression of CLC-3 in SGC-7901 cells, and the increase effect could be reversed by the PARP1 knockdown. **l**, **m** The proliferation and clonogenicity of SGC-7901 cells were promoted by XRCC5 overexpression, and the promotion effects were reversed by PARP1 knockdown (*n* = 3). *P < 0.05, **P < 0.01

The significant association between CLC-3 and XRCC5 revealed in cellular experiments and clinical outcomes led us to further examine their underlying molecular mechanisms. ChIP assays indicated that the binding of XRCC5 to the CLC-3 DNA in SGC-7901 cells was suppressed after XRCC5 knockdown (Fig. 5a). Dual-luciferase reporter assays revealed that knockdown of XRCC5 impaired the promoter activities of the pGL4.10-CLC-3 − 248 and pGL4.10-CLC-3 − 538 reporter plasmids in SGC-7901 cells (Fig. 5b). We then detected RNA expression of CLC-3 in established stable cell lines. The RNA level of CLC-3 was inhibited after XRCC5 knockdown and increased after XRCC5 overexpression, validating that the expression of CLC-3 was regulated by XRCC5 at the transcriptional level (Fig. 5c). Based on the primary related pathway of CLC-3 indicated above, we assessed whether the PI3K/Akt signaling pathway was inhibited after XRCC5 knockdown. The results demonstrated that knockdown of XRCC5 reduced the levels of key targets in the PI3K/Akt signaling pathway by downregulating CLC-3 in SGC-7901 cells (Fig. 5d).

Next, to identify the interaction partners of XRCC5 in regulating the expression of CLC-3, we performed immunoprecipitation (IP) combined with mass spectrometry (MS) in nuclear protein extracts of SGC-7901 cells with anti-XRCC5 antibodies. The MS results showed that 12 proteins were potential candidates for interaction with XRCC5, of which PARP1 was found to have the highest confidence score and coverage percentage (Fig. 5e). The best peptide-spectrum sequences of PARP1 and XRCC5 were shown in Fig. 5f, g. Next, the interaction between XRCC5 and PARP1 was confirmed by Co-IP in stable SGC-7901 cells with XRCC5 overexpression (Fig. 5h). To certify whether PARP1 also bound to the CLC-3 promoter, we pulled down the nuclear protein/DNA complex in GC cells using a CLC-3 promoter probe or NSP and validated their binding by WB (Fig. 5i). In addition, the subcellular localization of XRCC5 and PARP1 was examined by confocal microscopy analysis. The obtained images indicated that XRCC5 (green) and PARP1 (red) were both primarily expressed in the nucleus, and the co-localization of XRCC5 and PARP1 was observed in the nucleus (yellow) (Fig. 5j). The effect of PARP1 interacting with XRCC5 on CLC-3 expression was then explored. Overexpression of XRCC5 increased the expression of CLC-3 in SGC-7901 cells, and the increase effect could be reversed by PARP1 knockdown (Fig. 5k). Functionally, the promotion effects of XRCC5 overexpression on cell proliferation and clonogenicity were also reversed by PARP1 knockdown (Fig. 5l, m). Overall, the results indicated that the expression and function of CLC-3 were regulated at the transcriptional level by XRCC5 interacting with PARP1.

### The expression and function of CLC-3 were regulated by XRCC5 in vivo

**Fig. 6:**
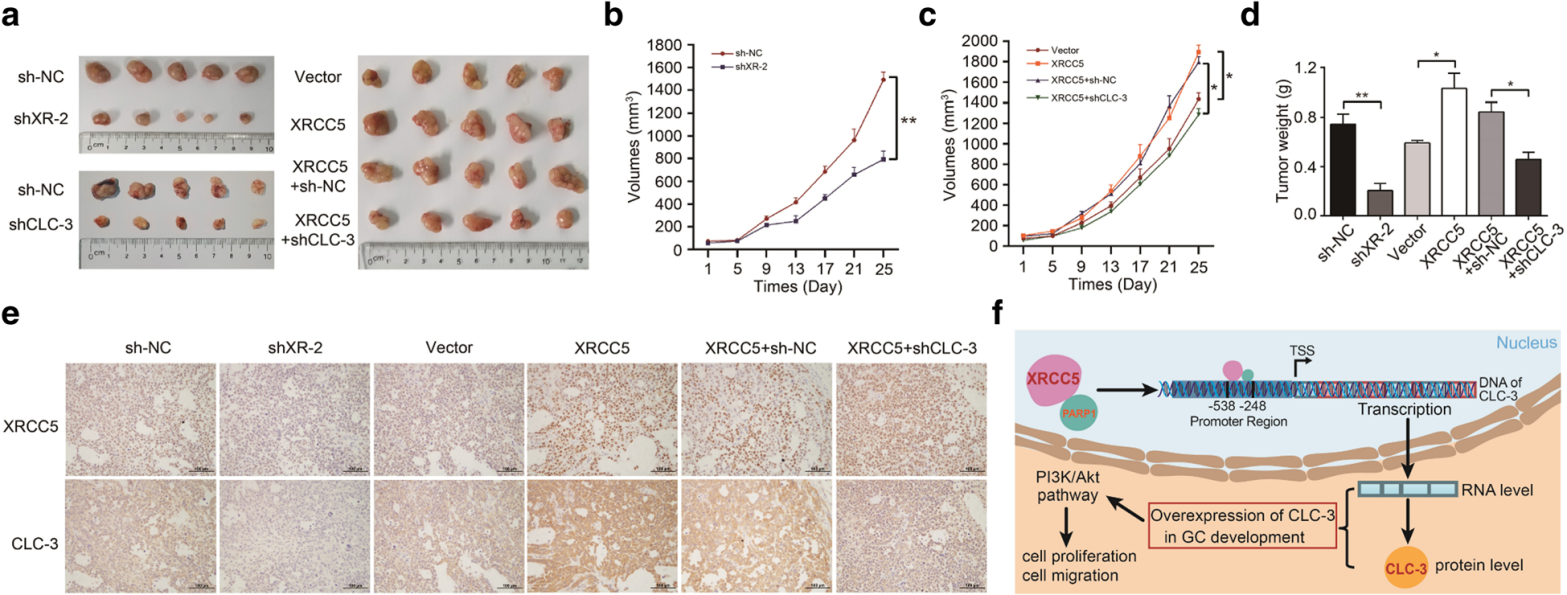
The expression and function of CLC-3 were regulated by XRCC5 in vivo. Nude mice were subcutaneously injected with SGC-7901 cells. **a** Images of GC tumor xenografts from each mouse (*n* = 5 mice/group). **b**, **c** Tumor volumes were recorded and analyzed. **d** Tumor weights were evaluated. **e** The expression of XRCC5 and CLC-3 in tumor tissues was analyzed by IHC staining. **f** Proposed model for the relationship between CLC-3 and XRCC5 in GC development. *P < 0.05, **P < 0.01

The association between XRCC5 and CLC-3 was also investigated in mouse xenograft models. SGC-7901 cells were subcutaneously injected into the left flank of nude mice. Tumor volumes were measured and recorded every 4 days. After approximately 4 weeks, the tumor xenografts were harvested, weighed, and processed for IHC staining. Our observations revealed that tumor growth was inhibited after XRCC5 knockdown or CLC-3 knockdown. Conversely, overexpression of XRCC5 promoted tumor growth and the promotion effect could be reversed by CLC-3 knockdown **(**Fig. 6a–d**)**. In addition, the expression of CLC-3 and XRCC5 in tumor tissues presented the same variation trend as tumor growth (Fig. 6e). These in vivo results were consistent with our in vitro observations and confirmed that CLC-3 could be regulated by XRCC5 in vivo.

## Discussion

GC is one of the most common tumors and continues to be a serious public health problem in the clinic. To date, the prognosis of advanced GC patients remains poor. Consistently, the available targeted therapy clinical trials only target HER2 (trastuzumab) and VEGFR2 (ramucirumab) in advanced GC patients [25]. Therefore, there is a need to explore more potential biomarkers of GC for therapeutic purposes. Chloride channels are a new class of membrane proteins that are aberrantly expressed in multiple tumor types. In addition to regulating various aspects of cancer cell behavior, chloride channels may constitute promising cancer biomarkers. However, few studies have focused on exploiting chloride channels for clinical purposes in GC. In this study, we first found that CLC-3, a member of the voltage-gated chloride channel superfamily, was overexpressed in human GC tissues and GC cell lines, suggesting a possible pivotal role of CLC-3 overexpression in GC development. Importantly, high expression of CLC-3 predicted poor prognosis in GC patients, demonstrating that overexpression of CLC-3 was a poor prognostic biomarker for GC.

CLC-3 is a crucial exchange transporter in plasma membranes and intracellular vesicles. Recently, the study of CLC-3 in cell metastasis and proliferation has attracted much attention [26–28]. Nonetheless, as a potential prognostic biomarker, the role of CLC-3 in digestive tract cancers is rarely reported, including GC. In this study, the primary biological functions of CLC-3 were identified as cell proliferation and migration, which were identical to the clinicopathological characteristics analysis of CLC-3 expression in GC patients, indicating that overexpression of CLC-3 in GC acted as a potential tumor-promoting factor by facilitating cell proliferation and migration. In addition, we found that the PI3K/Akt signaling pathway, a critical pathway mainly implicated in cell proliferation and migration [29, 30], was inhibited after the CLC-3 knockdown. This result was in accord with our previous finding indicating that the PI3K/Akt signaling pathway might be the downstream signaling pathway of CLC-3 [31]. So, we focused on the PI3K/AKT pathway rather than other pathways, and we hypothesized that CLC-3 might regulate cell proliferation and migration via this pathway. Accordingly, as a prognostic biomarker for GC, CLC-3 also plays important roles in vitro. Investigating the molecular mechanism of CLC-3 overexpression in GC development is needed.

To further explore the molecular mechanism of CLC-3 overexpression in GC, we studied the basic RNA expression of CLC-3 in cell lines. Elevated RNA level was also observed in GC cell lines, suggesting that a specific transcriptional regulatory mechanism of CLC-3 overexpression might exist in GC. With the regulatory events often occurring at gene promoters, we speculated that some tumor-specific cellular factors might bind specifically to the CLC-3 promoter to upregulate CLC-3 expression. Therefore, an optimal promoter probe was synthesized to pull down CLC-3 promoter-binding proteins [32, 33]. Our study demonstrated that XRCC5 bound the CLC-3 promoter, and increased combination was observed in GC cells, suggesting that this increased binding might be a promoting factor of CLC-3 overexpression. The novel finding of this study was the identification of XRCC5 as a CLC-3 promoter-binding protein in GC cells. To provide valuable clinical outcome prediction information, we then examined the expression of CLC-3 and XRCC5 in GC patients. Similar to most other types of tumors, the expression of XRCC5 in GC was also significantly increased [22–24]. Moreover, the expression of CLC-3 and XRCC5 presented the same variation trend, which was identified by their positive correlated expression in GC tissues. The survival analysis indicated that high expression of XRCC5 predicted poor prognosis in GC patients, prompting that XRCC5 might be a tumor-promoting factor in GC development. Importantly, the patients with high expression of XRCC5 and CLC-3 had the worst prognosis, revealing the synergistic effect of XRCC5 and CLC-3 on GC progression. Cox regression analysis further demonstrated that both CLC-3 and XRCC5 were prognostic factors of the overall survival in GC patients and that double detection of CLC-3 and XRCC5 could provide precise information for predicting the prognosis of GC patients. These findings indicate that the expression of CLC-3 is elevated in GC tissues in response to increased XRCC5 levels, and double targeting of CLC-3 and XRCC5 may provide more useful therapeutic potential for GC treatment.

As the regulatory subunit of the DNA-dependent protein kinase complex DNA-PK, XRCC5 is associated with the development of tumors such as lung cancer, breast cancer, and bladder cancer [34–36]. Functionally, XRCC5 acts as a double-edged sword by inhibiting or promoting tumor progression in different tumor types [37, 38]. However, little is known about the role of XRCC5 in GC. Clinicopathological characteristics analysis suggested that XRCC5 might promote the proliferation and invasion of GC cells. In vitro, the primary biological functions of CLC-3 were suppressed after XRCC5 knockdown and promoted after XRCC5 overexpression, reconfirming the tumor-promoting action of XRCC5. The rescue models with CLC-3 overexpression and reverse models with CLC-3 knockdown further certified that CLC-3 was the molecular target of XRCC5. Collectively, these results indicate that the expression and function of CLC-3 are regulated by XRCC5 in vitro and that XRCC5 is a tumor-promoting factor in GC. However, XRCC5 may not be the only regulator of CLC-3, and other molecular regulators can also exist in GC cells.

The above results only illustrated that XRCC5 bound to the CLC-3 promoter in the nucleus. To further verify the molecular mechanisms underlying the interaction between CLC-3 and XRCC5, ChIP assays and luciferase assays were performed in SGC-7901 cells with XRCC5 knockdown. We proved that knockdown of XRCC5 suppressed the binding of XRCC5 to the CLC-3 DNA and impaired the promoter activity of the pGL4.10-CLC-3 − 248 and pGL4.10-CLC-3 − 538 reporter plasmids, which indicated that the potential binding site might be located between − 248 and − 538. Furthermore, the RNA level of CLC-3 was inhibited by XRCC5 knockdown and increased by XRCC5 overexpression, validating that the expression of CLC-3 was regulated by XRCC5 at the transcriptional level. Next, XRCC5 knockdown inhibited the levels of key targets in the PI3K/Akt signaling pathway by downregulating CLC-3, confirming that the observed effects of XRCC5 on proliferation and migration were reflected at the functional level of CLC-3. Previous studies have indicated that XRCC5 binds the promoter region of genes such as pS2, FAS, and COX-2, thus regulating gene transcription. The XRCC5-interacting proteins identified in these genes’ promoter regions include DNA-PK, XRCC6, PARP-1, topoisomerase IIβ, PP1, and p300 [19, 20, 37]. Therefore, we tested whether XRCC5 bound to the CLC-3 promoter by interacting with other proteins. PARP1, also known as poly (ADP-ribose) polymerase 1, was discovered as a potential candidate for interaction with XRCC5 in the nucleus. Served as a transcription factor [39], PARP1 is essential for many cellular processes, including maintenance of genomic integrity, chromatin dynamics, and transcriptional regulation [40]. To ascertain whether PARP1 also bound the CLC-3 promoter, we pulled down the nuclear protein/DNA complex in GC cells using a CLC-3 promoter probe and validated their direct binding. We therefore speculated that PARP1 should also be identified in Fig. 2e. Nevertheless, the upper differential protein band (at almost 120 kDa) was not analyzed by MS. Previous studies have reported that PARP1 often forms regulatory complexes with other proteins and then regulates the expression of genes, including CCND1, CCN2, and NF-κB [41–43]. Indeed, the co-localization of XRCC5 and PARP1 was observed in the nucleus, indicating that XRCC5 and PARP1 formed a regulatory complex in the nucleus. It has been reported that PARP1 is overexpressed in GC and that PARP1 knockdown significantly attenuates the proliferation of GC cells [44, 45]. Therefore, we preliminarily explored the interaction effect of PARP1 and XRCC5 on CLC-3 expression and cell proliferation. The results indicated that the promotion effect of XRCC5 overexpression on the CLC-3 expression and cell proliferation was partly reversed by the PARP1 knockdown, which suggested that PARP1 might act as a positive regulatory factor for CLC-3 in GC cells. These findings prove that knockdown of XRCC5 suppresses the binding of XRCC5 to the CLC-3 promoter and subsequent promoter activity, thus regulating CLC-3 expression at the transcriptional level by interacting with PARP1. Currently, there are ongoing clinical trials of many PARP1 inhibitors aimed at DNA binding and transcriptional activity [46–48], and one PARP inhibitor, olaparib, has been approved by the FDA to treat ovarian cancer patients with BRCA genes mutations [49], providing new prospects for the application of this inhibitor in future studies.

The relationship between CLC-3 and XRCC5 was also investigated in mouse xenograft models. The results were consistent with our in vitro data and showed that CLC-3 could be regulated by XRCC5 in vivo. Here, we propose a model for the relationship between CLC-3 and XRCC5 in GC development (Fig. 6f). Overexpression of CLC-3 is a poor prognostic biomarker for GC, and CLC-3 may regulate cell proliferation and migration via the PI3K/AKT signaling pathway. The regulatory mechanism of CLC-3 overexpression in GC is that XRCC5 binds the CLC-3 promoter region and affects subsequent promoter activity, thus regulating CLC-3 expression at the transcriptional level by interacting with PARP1.

## Conclusions

In summary, this study has illustrated that overexpression of CLC-3 is regulated by XRCC5 and is a poor prognostic biomarker for gastric cancer. Double targeting CLC-3 and XRCC5 may provide promising therapeutic potential for GC treatment.

## Additional file

Additional file 1: **Table S1.** The truncated promoter regions of CLC-3 were designed with primer pairs as follows. (DOCX 29 kb)

## Abbreviations

ANT: Adjacent normal tissues
BSA: Bovine serum albumin
cDNA: Complementary DNA
ChIP: Chromatin immunoprecipitation
CI: Confidence interval
CLC-3: Chloride channel-3
Co-IP: Co-immunoprecipitation
DAB: 3,5-Diaminobenzidine
DAPI: 4,6-Diamidino-2-phenylindole
DGE: Differential gene expression
DNA-PK: DNA-dependent protein kinase
FBS: Fetal bovine serum
GC: Gastric cancer
GO: Gene Ontology
HR: Hazard rate
IF: Immunofluorescence
IHC: Immunohistochemistry
IP: Immunoprecipitation
KEGG: Kyoto Encyclopedia of Genes and Genomes
MS: Mass spectrometry
MTS: 3-(4,5-Dimethylthiazol-2-yl)-5-(3-carboxymethoxyphenyl)-2-(4-sulfophenyl)-2H-tetrazolium
NSP: Nonspecific probe
OD: Optical density
PAGE: Polyacrylamide gel electrophoresis
PARP1: Poly (ADP-ribose) polymerase 1
PBS: Phosphate-buffered solution
PCR: Polymerase chain reaction
PVDF: Polyvinylidene fluoride
qRT-PCR: Quantitative real-time polymerase chain reaction
RPMI: Roswell Park Memorial Institute
SDS: Sodium dodecyl sulfate
SRA: Sequence Read Archive
WB: Western blot
XRCC5: X-ray repair cross-complementing 5
