# Supplementary material for: Overexpression of CLC-3 is regulated by XRCC5 and is a poor prognostic biomarker for gastric cancer
Source: J Hematol Oncol. 2018 Sep 14;11:115. doi: 10.1186/s13045-018-0660-y (PMC6137920; doi:10.1186/s13045-018-0660-y)
Supplement: Supplementary file 1 — Table S1. The truncated promoter regions of CLC-3 were designed with primer pairs as follows. (DOCX 29 kb) [file 13045_2018_660_MOESM1_ESM.docx]

**Table S1** The truncated promoter regions of CLC-3 were designed with primer pairs as follows.

| **Truncated regions** | **Forward (5’-3’)** | **Reverse (5’-3’)** |
| --- | --- | --- |
| **-1089 ~ +226** | TGGCAAGACCAGGTCATAT | AACCTGCTTCTGCCCAAAAT |
| **-972 ~ +226** | CACGGGTCTTAATTCTGCC | AACCTGCTTCTGCCCAAAAT |
| **-822 ~ +226** | AGCACCTTGAACACGGGAT | AACCTGCTTCTGCCCAAAAT |
| **-538 ~ +226** | GCTTTTGGTGCTGGTTTAT | AACCTGCTTCTGCCCAAAAT |
| **-248 ~ +226** | CTAATCGCTAATGACAGGC | AACCTGCTTCTGCCCAAAAT |
| **+73 ~ +226** | CCCCTTTCCCAGTGTTCTA | AACCTGCTTCTGCCCAAAAT |
| **The promoter region of CLC-3（the transcriptional start site is showed in the red rectangle ）** | | |
| GGCTGGCAAGACCAGGTCATATTTAATATTCATGAGGCACAAGATATACAACACGCACTTAATATTCATTAATCAGTATATATTCATAATATACTAAATATACAGGAGATATTAAACGTTCACGGGTCTTAATTCTGCCACCCAGTGGAGACACTCAAAAGTGATCTCGCCAAAGAAATGCAAAACCTGTACCTGCCCACCAGGCTATTAGCATGCTACTGCTGTGGCCACCATTCTTTCACTGCATCGCCCTGCCCTGCATCGGAATATAGCACCTTGAACACGGGATCAGGTTTACTGAATTCTGTGACCCACGCTGGGAGCTCAATAAATGTTTGCTAAACGGAAATGAACCCAATTCAGACCGTGTAGAAACCCCAGATTATATCTTTTCTCCAAACATTCAATTTCCAAGCAGTATTTTTGAAAATGCGTGATGGCTAAATTCGAGTTATCCTTTTTGTATGTATGTTAGGCACACAACCGGCGATGACATACAAGGCGAGGTGACACCACTGCACTCCCAGCCCGCACGTCACCTATAAGCACAGGGGGCTTTTGGTGCTGGTTTATTTTTGAAGGCTGGTACTTCTCTTCGATCTAAGTTTTAGGAACCTGTGCTCGTCGACGGCTGCCAATACTTTTTATTTTCAAGGCTGACGCTCGCCAGAGTTTCACAAGCTGCTCCCGGACGCTGTGGTCTCTCGCTAGAGTGGATCCGAACTCTGCGTGACGCTGGGAACGGCCAGCGTCTGTTGCCTCACGAAAACATCTCCCACAAGGTGGCCGCCTCGCGCGAGGTCAGCAGCAGGGCAAACAGGGATGCGTGAGTGACTGACGTGAGCTAATCGCTAATGACAGGCTTCACAGCCCCGGCGGCCCTCAGAGCCAGGCACCTCGCACCCCTCCTCCTCCTCGAGACCCAGTCCCGGCCCGCCCACCGTTCCCACGCCGCCCAATAGGCGCCGACGCTACTCAGCGAGTGGCCGGAGGAGGAGGTGTTGGGACGGCCGGACGCTGAACGGCCGTTCACGTCAACGTAGTGACGTCACGCGTCGACGCTGGGGCGTACCTTTCGGGCTCCTGACTCCTGCCGCTTCTCTTCCCCTTCCGTGGGTCAGGGCCGGTCCGGTCCGGAACCTGCAGCCCCTTTCCCAGTGTTCTAGTTCGCCCGTGACCCGGAATAATGAGCAAGGAGGGTGTGGTGGGTTGAAAGCCATCCTACTTTACTCCCGAGTTAGAGCATGGATTCAGTTTTAGTCTTAAGGGGGAAGTGAGATTGGAGATTTTTATTTTTAATTTTGGGCAGAAGCAGGTTGACTC | | |
